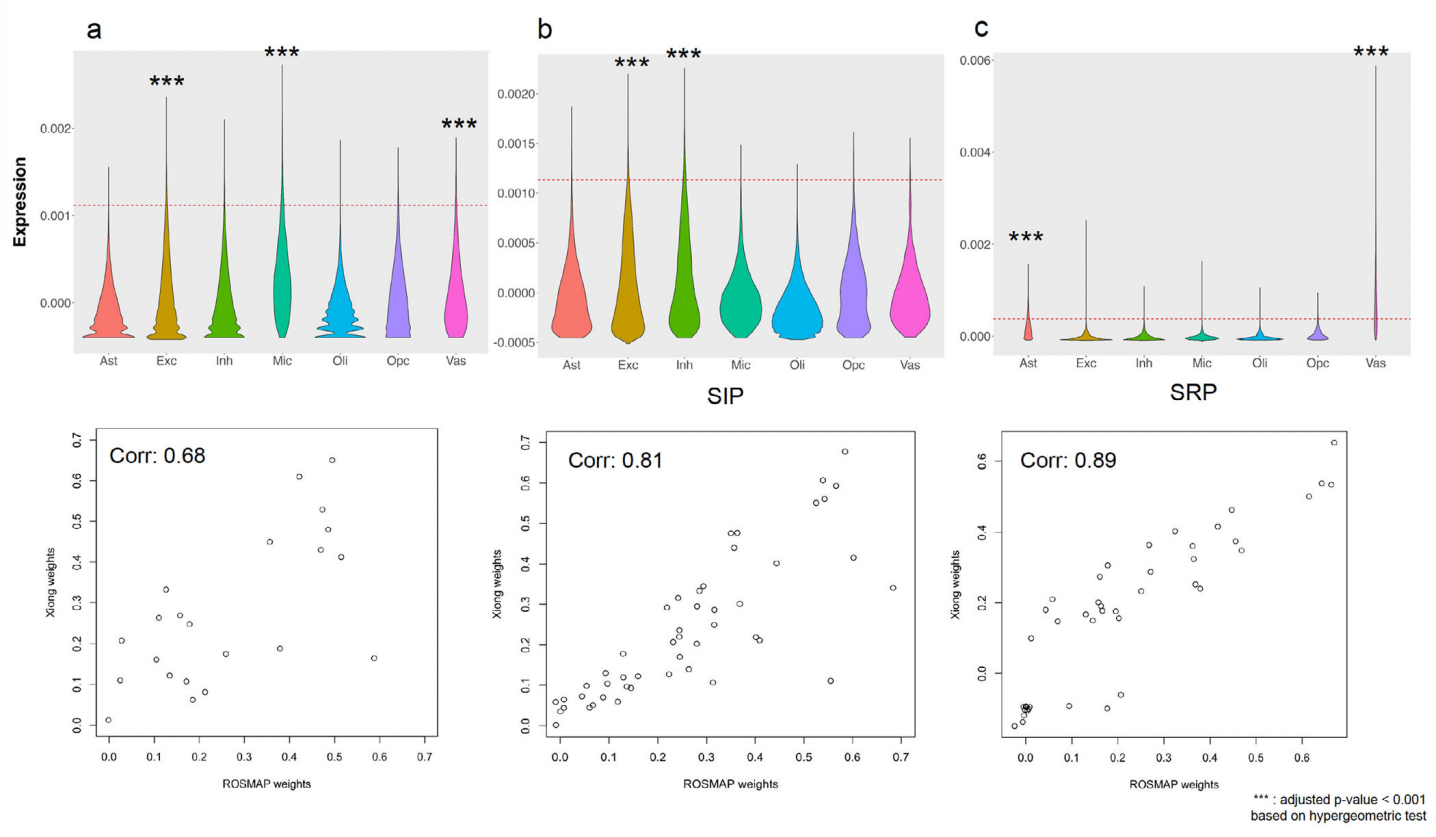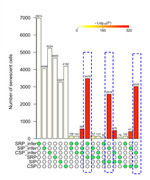# Tracing senescence in the Alzheimer’s brain through an eigengene‐based analysis of snRNA‐Seq data

**DOI:** 10.1002/alz.090422

**Published:** 2025-01-03

**Authors:** Shiva Kazempour Dehkordi, Habil Zare, Miranda E. Orr

**Affiliations:** ^1^ University of Texas Health Science Center at San Antonio, San Antonio, TX USA; ^2^ The University of Texas Health Science Center at San Antonio, San Antonio, TX USA; ^3^ Wake Forest University School of Medicine, Winston‐Salem, NC USA

## Abstract

**Background:**

Cellular senescence, which can cause significant changes in morphology, metabolism, and function, is a key contributor to aging and diseases including Alzheimer’s Disease (AD). Accurate biomarker identification is essential for detecting senescent cells. Our research aims at defining gene signatures that encapsulate senescence complexity in the brain. Our previous study, highlighted *CDKN2D*/p19 as a reliable senescence marker in tau neuropathology. This study advances our approach using a new snRNA‐Seq dataset.

**Method:**

We evaluated snRNA‐Seq data from 92 postmortem brains (PMID: 37774680) to validate senescent cell types, utilizing the transcriptional profiles of around 414,000 cells. We used an eigengene‐based approach, where an eigengene is a weighted average expression of genes that summarizes biological signatures with minimal loss of information (PMID: 28298217). Using established weightings from our prior research (PMID: 35531351), we inferred eigengenes indicative of senescence in this dataset. We also recalculated eigengenes for key senescence pathways ‐ the canonical senescence pathway (CSP) which reflects cell cycle arrest, the senescence initiator pathway (SIP) indicating cellular damage and stress, and the senescence response pathway (SRP) representing the toxic senescence‐associated secretory phenotype (SASP) ‐ and assessed the overlap of senescent cells using statistical tests.

**Result:**

Using eigengenes inferred from our previous study, we confirmed that excitatory neurons significantly expressed CSP and SIP signatures in the new dataset. Additionally, microglia and vascular cells in CSP, inhibitory neurons in SIP, and astrocytes and vascular cells in SRP showed significant enrichment (Fig. 1a‐c). Recalculation of eigengenes supported these findings by returning the enrichment of exact cell types within each pathway, demonstrating the method’s robustness in identifying senescent cell types. Furthermore, a substantial number of cells showed overlapping senescence signatures across the CSP, SIP, and SRP (Fig. 2), confirming consistency between inference and recalculated eigengenes.

**Conclusion:**

Our findings validate a computational strategy for detecting senescent cells via eigengene analysis. Consistent identification of senescent cell signatures between inferred and recalculated eigengenes across neural and vascular cells confirms the accuracy of our methods. This approach may direct AD therapeutic development by pinpointing precise senescent cell targets, and uncover new biomarkers of brain cell senescence.